# Smartphone Photogrammetry as a Tool for Pes Planus Assessment: Reliability and Agreement with Radiographic Measurements

**DOI:** 10.3390/diagnostics16020253

**Published:** 2026-01-13

**Authors:** Emre Mucahit Kartal, Gultekin Taskıran, Hakan Cetin, Murat Yuncu, Mehmet Barıs Ertan, Ozkan Kose

**Affiliations:** 1 Department of Orthopedics and Traumatology, Antalya City Hospital, 07070 Antalya, Turkey; emre_mucahit_kartal@hotmail.com (E.M.K.); gultekin3838@outlook.com (G.T.); 2 Department of Orthopedics and Traumatology, Sanlıurfa Mehmet Akif Inan Education and Research Hospital, 63040 Sanlıurfa, Turkey; dr.hakancetin@outlook.com; 3 Orthopedics and Traumatology Clinic, Elmalı State Hospital, 07716 Antalya, Turkey; m-yuncu@yandex.com; 4 Orthopedics and Traumatology Clinic, Private Medikum Hospital, 07350 Antalya, Turkey; mehmetbarisertan@gmail.com; 5 Department of Orthopedics and Traumatology, University of Health Sciences, Antalya Education and Research Hospital, 07100 Antalya, Turkey

**Keywords:** pes planus, calcaneal pitch angle, smartphone photography, photogrammetry, reliability, diagnostic accuracy

## Abstract

**Background/Objectives:** The purpose of this study was to evaluate the reliability and diagnostic accuracy of smartphone-based photogrammetry for the assessment of pes planus and to determine its agreement with standard radiographic measurements. **Methods:** This prospective diagnostic study included 100 skeletally mature patients (50 males, 50 females; mean age 43.4 years) who underwent standardized lateral weight-bearing foot radiographs and smartphone-based foot photography. The calcaneal pitch angle (CPA) was measured on radiographs, and a corresponding photographic arch pitch angle (P-APA) was measured from standardized smartphone photographs using digital software (Angle Meter iOS v1.9.8). Three independent observers performed each measurement twice. Inter- and intra-observer reliability was assessed using intraclass correlation coefficients (ICC). Agreement between methods was evaluated with Pearson correlation, Lin’s concordance correlation coefficient (CCC), Bland–Altman analysis, and Deming regression. Receiver operating characteristic (ROC) analysis was performed to determine the diagnostic accuracy of calibrated P-APA, with the radiographic threshold of 18° serving as the reference standard for pes planus classification. **Results:** All measurements demonstrated excellent intra- and inter-observer reliability (ICC ≥ 0.900). P-APA values were systematically higher than radiographic values (31.8° ± 4.3 vs. 21.8° ± 5.5; *p* < 0.001). A strong correlation was observed between the two methods (r = 0.799, *p* < 0.001), but concordance was poor (CCC = 0.222). Bland–Altman analysis revealed a mean bias of +10.1° with wide limits of agreement (3.8° to 16.4°). Deming regression yielded the calibration equation Radiographic CPA = (P-APA × 1.371) − 21.883. ROC analysis of calibrated values yielded an AUC of 0.885 (95% CI, 0.820–0.951), with an optimal cutoff of 22.8° (sensitivity, 100%; specificity, 61.1%), corresponding to 32.6° on the uncalibrated photographic scale. **Conclusions:** Conventional weight-bearing radiography remains the reference standard for diagnosis and clinical decision-making in pes planus. The smartphone-derived photographic arch pitch angle is a non-equivalent surrogate measure that shows substantial systematic bias and limited agreement with radiographic calcaneal pitch, and therefore cannot replace weight-bearing radiographs. Smartphone photogrammetry may be used only as a complementary tool for preliminary screening or telemedicine support; any positive or equivocal findings require radiographic confirmation.

## 1. Introduction

Pes planus, commonly referred to as flatfoot, is a musculoskeletal condition characterized by the collapse of the medial longitudinal arch of the foot [1]. It may present as flexible or rigid and is frequently associated with altered lower limb biomechanics, pain, gait abnormalities, and functional limitations. In addition, pes planus has been linked to a variety of foot and ankle pathologies, including hallux valgus, metatarsalgia, plantar fasciitis, and an increased risk of lower-extremity injuries [2]. Its prevalence varies widely across populations and age groups, ranging from asymptomatic flexible flatfoot in children to acquired rigid deformities in adults, highlighting the clinical importance of accurate diagnosis and assessment [3].

Traditionally, the diagnosis of pes planus relies on clinical examination and weight-bearing radiographic measurements, which are considered the gold standard for evaluating arch morphology and alignment [4,5,6]. Radiographic parameters, such as Meary’s angle and calcaneal pitch, provide valuable insights into osseous alignment [7]. Beyond radiography, several conventional approaches have been proposed to evaluate foot arch height. Footprint-based indices such as the Arch Index, Clarke’s angle, Staheli, and the Chippaux–Smirak index have been widely used in both clinical and epidemiological research [8,9,10,11]. These techniques are inexpensive, non-invasive, and easily repeatable; however, their accuracy compared to radiographic methods has been a topic of debate. Some studies report moderate correlations, while others question the diagnostic validity of these findings [12,13,14].

Recent advances in mobile technology have introduced novel opportunities for musculoskeletal assessment. The widespread availability of smartphones with high-resolution cameras has enabled the use of two-dimensional (2D) photogrammetry as a low-cost, accessible method for evaluating foot morphology [15]. Standardized photographs of the foot can be analyzed to calculate angles and indices analogous to those used in radiographic analysis. Initial studies suggest that these methods could be promising, particularly in the context of telemedicine and digital health applications [16,17].

Nevertheless, despite the growing interest in digital solutions, a gap remains in the literature: only a limited number of studies have directly compared smartphone-based photographic foot measurements with radiographic standards in the evaluation of pes planus [18,19,20]. Addressing this gap is crucial to determining whether smartphone photography can serve as a reliable and valid diagnostic alternative. Based on these considerations, we hypothesized that pes planus can be reliably assessed using smartphone photography, with measurements showing acceptable agreement with radiographic evaluations. Therefore, this study addresses the lack of radiograph-referenced validation for smartphone photogrammetry. A standardized smartphone photography method is presented to measure a photographic arch pitch angle (P-APA) as an external surrogate of medial arch geometry, and to develop a radiograph-referenced calibration for estimating radiographic CPA. Reliability, agreement, and diagnostic performance were evaluated to support triage and telemedicine follow-up when radiographs are not readily available.

## 2. Materials and Methods

### 2.1. Study Design and Patient Selection

This prospective diagnostic study was conducted at a tertiary orthopedic outpatient clinic between June and July 2025. Skeletally mature patients presenting with foot and ankle complaints who were clinically indicated for lateral full–weight-bearing foot radiographs were screened for eligibility. Eligible participants were those undergoing lateral foot radiography in a full weight-bearing position. Patients were excluded if they had a history of foot or ankle fractures, prior foot or ankle surgery, or congenital foot deformities, or if they declined to participate. Written informed consent was obtained from all participants before enrollment. The study protocol was approved by the Institutional Review Board (Approval Date/Issue: 29 May 2025/9-15), and all procedures were conducted in accordance with the Declaration of Helsinki.

### 2.2. Sample Size Calculation

The sample size was calculated a priori to detect a clinically relevant bias of 2° between smartphone-based and radiographic measurements of the calcaneal pitch angle (CPA). An equivalence framework for the mean difference between methods (two one-sided tests) was adopted, with an equivalence margin of ±2° and a significance level of 0.05. The standard deviation of the differences was assumed to be 6.2°, based on the upper end of the reported standard deviations for CPA and related arch-height angles in a previous radiographic study [21]. Under these assumptions and with a statistical power of 90%, the required sample size was estimated at approximately 83 patients, each providing one smartphone-based and one radiographic measurement. However, to enhance the study’s statistical power and reliability, 100 patients were prospectively enrolled. This prespecified equivalence assessment targeted the mean bias; interchangeability at the individual level was additionally evaluated using Bland–Altman limits of agreement.

### 2.3. Radiographic Evaluation and Measurements

**Figure 1:**
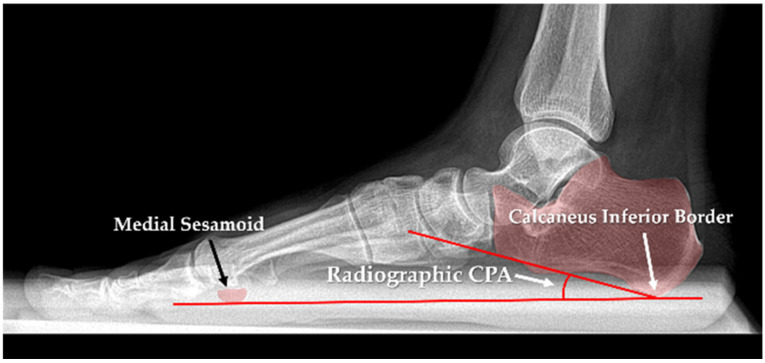
Radiographic measurement of the CPA. The CPA was determined on a lateral weight-bearing radiograph by drawing a baseline line between the inferior border of the medial sesamoid and the inferior border of the calcaneus. A second line was placed parallel to the inferior surface of the calcaneus. The angle formed between these two lines represents the radiographic CPA.

Standard lateral weight-bearing foot radiographs were obtained for each participant using institutional imaging protocols. Images were acquired with the patient in a full single-leg stance to ensure physiological foot loading. Radiographic measurements were performed using a digital workstation picture archiving and communication system (PACS). Radiographic CPA was measured on a lateral weight-bearing radiograph as the angle between a reference line drawn from the inferior calcaneal surface to the medial sesamoid and the line tangential to the inferior surface of the calcaneus, as illustrated in Figure 1. A CPA of less than 18° was considered indicative of pes planus [22,23,24], and this commonly used radiographic criterion was adopted as the reference standard in the present study.

### 2.4. Foot Photography and Digital Measurements

**Figure 2:**
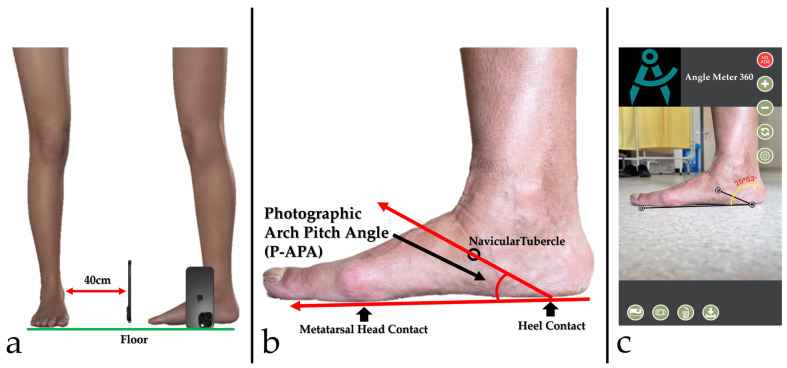
Standardized smartphone photography setup and measurement of the photographic arch pitch angle (P-APA). (**a**) Lateral foot photographs were acquired at a fixed distance of 40 cm, with the smartphone placed directly on the floor in an upright position (no tripod), and the camera lens oriented perpendicular to the floor to minimize tilt. Images were obtained without digital zoom. (**b**) Anatomical landmarks used for photographic arch pitch angle (P-APA) measurement: the baseline reference line was drawn between the plantar contact points of the first metatarsal head and the calcaneal tuberosity, and a second line extended from the calcaneal contact point to the navicular tubercle. (**c**) Example of digital measurement performed with the Angle Meter 360° application, demonstrating the calculated photographic arch pitch angle (P-APA).

Each participant underwent standardized foot photography using an iPhone 16 Pro Max (Apple Inc., Cupertino, CA, USA) smartphone. Before image acquisition, the navicular tubercle was palpated and marked using a visible, non-reflective skin-safe marker. Patients were instructed to stand barefoot in a single-leg stance to simulate maximal load on the tested foot. Photographs were taken from the medial side of the foot, with the camera held horizontally, perpendicular to the ground, and parallel to the foot’s longitudinal axis. Care was taken to ensure that the entire foot was captured within the frame. For all participants, photographs were obtained from a fixed distance of 40 cm. A tripod was not used; instead, the smartphone was placed directly on the floor in a stable position, maintaining a consistent setup throughout image acquisition. The device was positioned upright with the camera lens oriented perpendicular to the floor, thereby minimizing tilt. The camera lens height above the floor was the fixed lens height of the device in this upright position. Standardized alignment was ensured by using the same foot placement and stance instructions for all participants. Digital measurements were performed using the Angle Meter 360° smartphone application (developed by Alexey Kozlov, Apple Inc., Cupertino, CA, USA, available at https://apps.apple.com/us/app/angle-meter-360/id1393860479, accessed on 11 January 2026). The standardized photographs were imported into the application, which provides an on-screen goniometer function operating directly on the image. A baseline reference line was drawn between the plantar contact points of the first metatarsal head and the calcaneal tuberosity, simulating the ground plane. A second line was drawn from the calcaneal contact point to the navicular tubercle. The angle formed between these two lines was defined as the photographic arch pitch angle (P-APA) (Figure 2). The goniometer arms were positioned at predefined reference points/lines on the photograph, and the image was magnified as needed to allow more precise placement of the selected landmarks. The same measurement workflow was used for all participants. Because this construct is based on external plantar contact points and a marked navicular tubercle, it represents a surrogate measure against the radiographic CPA rather than the same anatomic angle. Therefore, P-APA should not be interpreted as a direct photographic replication of radiographic CPA.

### 2.5. Intra- and Interobserver Reliability

To assess measurement reproducibility, three orthopedic surgeons independently performed the photographic and radiographic evaluations. Each observer completed the measurements twice, with a minimum interval of 15 days between assessments. All evaluations were conducted in a blinded fashion; observers were unaware of previous results and those of the other evaluators.

Inter- and intra-observer reliability of the photographic and radiographic measurements was quantified using the intraclass correlation coefficient (ICC) with 95% confidence intervals (CI). Inter-observer reliability was estimated with ICC (2,1) (two-way random-effects, absolute agreement), and intra-observer reliability with ICC (3,1) (two-way mixed-effects, consistency). Reliability was interpreted as follows: <0.50, poor; 0.50–0.75, moderate; 0.75–0.90, good; and >0.90, excellent. Because all ICCs were ≥0.900 (excellent), subsequent analyses used the mean of the three raters’ two sessions for both the photographic arch pitch angle and the radiographic CPA (Table 1 and Table 2).

### 2.6. Statistical Analysis

Descriptive statistics, including means, standard deviations, and ranges, were calculated for radiographic CPA and photographic P-APA. Between-method agreement was assessed using Bland–Altman analysis; the mean difference (bias) and 95% limits of agreement (LoA) were computed to quantify agreement and the magnitude of individual-level differences. In addition, equivalence of the mean between-method difference was evaluated using a two one-sided tests (TOST) procedure with equivalence bounds of −2° and +2° and α = 0.05; equivalence was defined a priori as the 90% confidence interval for the mean difference lying entirely within the ±2° margin. A paired *t*-test was used to compare mean values between methods. Pearson correlation was calculated to describe the linear association between methods; however, validity and interchangeability were evaluated primarily using agreement-based approaches, including Lin’s concordance correlation coefficient and Deming regression. ROC analysis was performed to assess the ability of the calibrated photographic measure to discriminate pes planus using radiographic CPA < 18° as the reference standard; AUC was calculated, and the optimal cutoff was determined using Youden’s J index. For the selected cutoff, sensitivity, specificity, PPV, and NPV were calculated from a 2 × 2 contingency table, and 95% confidence intervals were computed using the exact binomial method. Because the calibration model and ROC analyses were performed on the same dataset, these estimates should be interpreted as derivation (exploratory) results, and no internal or external validation was applied. All analyses were performed using SPSS version 25.0 (IBM Corp., Armonk, NY, USA), and all tests were two-tailed with a significance level of *p* < 0.05.

## 3. Results

### 3.1. Demographic Characteristics of the Study Population

A total of 100 (50 males and 50 females) participants were included in the study. The mean age was 43.4 years (range, 16–75 years; SD ± 15.1). Of the study sample, two participants were 16 years old, and all remaining participants were 18 years or older. The mean height was 168.8 cm (range, 147–198 cm; SD ± 9.2), and the mean weight was 80.8 kg (range, 43–125 kg; SD ± 14.7). The average body mass index (BMI) of the cohort was 28.3 kg/m^2^ (range, 18.9–39.4; SD ± 4.4). Side distribution was nearly equal, with 48 right (48%) and 52 left (52%) feet. Twenty-eight (28%) patients had pes planus (radiographic CPA < 18°), and the remaining 72 (72%) patients had normal feet.

### 3.2. Between-Method Comparison and Agreement

**Figure 3:**
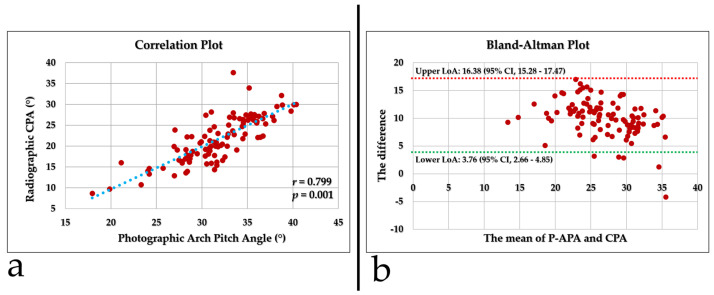
Correlation and agreement between P-APA and CPA. (**a**) Scatter plot showing the relationship between the P-APA and CPA, demonstrating a positive correlation (r = 0.799, *p* = 0.001). (**b**) Bland–Altman plot illustrating the difference between P-APA and CPA against their mean, with a mean bias of approximately 10° and 95% limits of agreement ranging from 3.76° to 16.38° (dashed lines; 95% CI for upper LoA 15.28–17.47 and for lower LoA 2.66–4.85).

P-APA measurements were systematically higher than radiographic CPA measurements (31.83 ± 4.30 vs. 21.77 ± 5.49, *p* < 0.001). The paired difference was 10.06 ± 3.3 (range, −4.2–16.9) degrees. In the prespecified TOST analysis, the equivalence hypothesis (±2°) was not met; the 90% confidence interval for the mean difference (9.51° to 10.61°) lay entirely outside the equivalence bounds. Pearson correlation analysis demonstrated a positive linear association between photographic and radiographic measurements (r = 0.799, *p* = 0.001) (Figure 3a). However, correlation only reflects the strength of linear association and does not necessarily indicate agreement between methods. Consistently, Lin’s concordance correlation coefficient revealed poor agreement (CCC = 0.222; 95% CI, 0.169–0.282), suggesting that despite the strong correlation, photographic measurements systematically overestimated radiographic values and lacked sufficient concordance. Bland–Altman analysis showed a positive mean bias of 10.06 (SD 3.3), with 95% limits of agreement (LoA) of 3.76 to 16.38; the 95% CI for the bias was 9.428–10.706, and the 95% CI for the LoA were 2.661–4.852 (lower) and 15.283–17.474 (upper). Thus, the expected 95% prediction interval for an individual photographic-radiographic difference corresponds to the LoA (3.76–16.38), indicating substantial systematic and random discrepancies that preclude interchangeability (Figure 3b).

### 3.3. Calibration of the Photographic to the Radiographic Angle

To derive a conversion from the photographic measure to the radiographic calcaneal pitch, we fitted a Deming regression (λ = 1), which accounts for measurement error in both methods. The model yielded an intercept of α = −21.883 (95% CI, −34.283 to −14.896) and a slope of β = 1.371 (95% CI, 1.150 to 1.756), indicating the presence of both constant and proportional bias. The resulting calibration equation was: Radiographic CPA = (P-APA × 1.371) − 21.883. This calibration equation was derived from the present dataset and has not been internally or externally validated.

Model performance on the study dataset was coefficient of determination (R^2^) = 0.529, root mean squared error (RMSE) = 3.528°, and mean absolute error (MAE) = 2.603°; the SD of residuals was 3.5°, corresponding to an approximate 95% prediction interval (PI) for a single new case of predicted value (ŷ) ± 6.9°. Collectively, these estimates indicate that while photographic measurements can be calibrated to radiographic values, a non-negligible prediction error remains at the individual level.

### 3.4. Diagnostic Accuracy and ROC Analysis of Calibrated Smartphone Measurements

**Figure 4:**
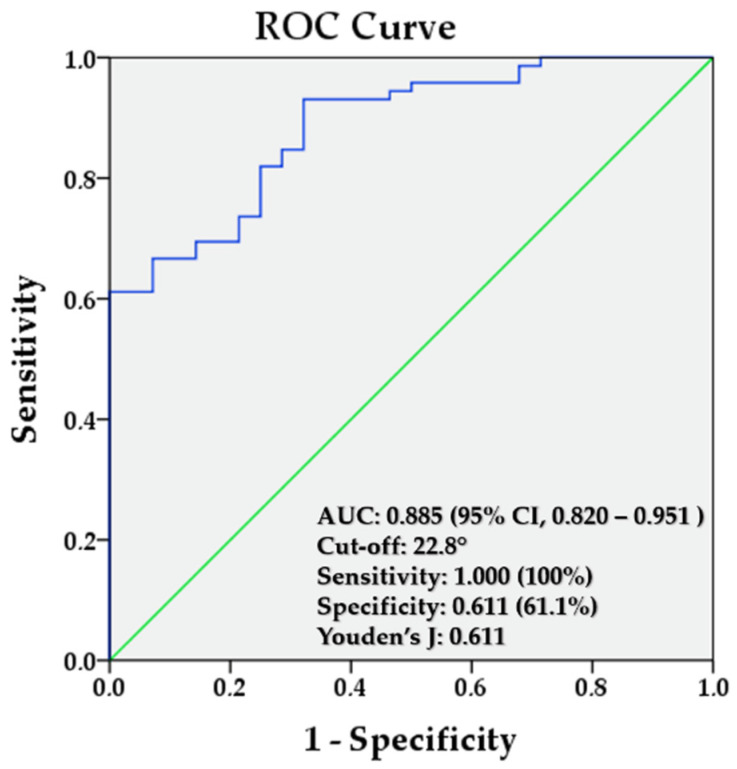
ROC curve analysis of the calibrated photographic arch pitch angle (P-APA) for estimating radiographic CPA.

For the radiographic assessment of the CPA, a threshold of 18° was used as the reference standard to classify feet as having pes planus or normal alignment. To establish a corresponding threshold for the calibrated P-APA, a receiver operating characteristic (ROC) analysis was conducted. The analysis demonstrated an area under the curve (AUC) of 0.885 (95% CI, 0.820–0.951). The optimal cutoff value, determined by Youden’s J index, was 22.8° (sensitivity = 1.000, specificity = 0.611), corresponding to approximately 32.6° on the uncalibrated photographic scale (Figure 4). The optimal cutoff value, determined by Youden’s J index, was 22.8° (Figure 4). Using this cutoff (test positive defined as calibrated value ≤ 22.8°), sensitivity was 100% (95% CI, 87.7–100.0) and specificity was 61.1% (95% CI, 48.9–72.4). The corresponding PPV was 50.0% (95% CI, 36.3–63.7) and the NPV was 100.0% (95% CI, 92.0–100.0). A 2 × 2 contingency table is provided in Table 3.

## 4. Discussion

In this study, it was shown that the P-APA calculated from standardized lateral foot photographs taken with a smartphone exhibits high reliability in the evaluation of pes planus (intraclass correlation coefficient [ICC] ≥ 0.90). The fact that both intra- and inter-observer repeatability were near-perfect demonstrates that the photographic measurement protocol yields consistent results. However, high reliability reflects repeatability and does not imply validity or agreement with the reference standard. It was determined that photograph-based arch pitch angle values systematically overestimated the radiographic CPA (by an average of 10°) and that both constant and proportional biases existed between the two methods. In the prespecified TOST analysis, equivalence within ±2° was not demonstrated, confirming that P-APA is not equivalent to radiographic CPA and should not be used as a direct substitute. Despite the strong correlation, the low concordance and wide Bland–Altman limits indicate that using photographic measurements directly in place of radiographic decision thresholds without calibration is prone to error. The calibration equation obtained with Deming regression (CPA = 1.371 × P-APA − 21.883) quantitatively compensates for this systematic offset, and the ROC analysis for pes planus discrimination using calibrated values yielded high diagnostic performance (AUC 0.885; best threshold 22.8°; sensitivity 100%, specificity 61.1%; corresponding to approximately 32.6° on the photographic scale). This suggests that the method may be considered a preliminary triage-oriented tool in similar clinical contexts; however, given the lack of independent validation and modest specificity, radiographic confirmation should be maintained, and the reported diagnostic performance should be interpreted cautiously. Importantly, our observed systematic offset is consistent with prior evidence that medial longitudinal arch morphology is not fully captured by a single angular descriptor: functional arch indices correlate more consistently with sagittal radiographic angles such as Meary’s (talo–first metatarsal) and the talo-horizontal angle, whereas calcaneal pitch alone may show weak or inconsistent association with arch height [25].

The reliability and validity profile of the photographic method are consistent with the literature, demonstrating convergence between external foot measurements and radiographic parameters. Numerous supporting studies in the literature indicate that two-dimensional photogrammetric techniques are reliable for diagnosing pes planus. McPoil and colleagues reported that changes in dorsal arch height measured with digital photographs during sitting-to-standing transitions showed high intra- and inter-observer reliability and high validity against a radiographic reference [26]. Similarly, Pohl and Farr reported that the arch height index calculated by the digital photograph method showed excellent agreement with values measured by a mechanical caliper (ICC > 0.86) and a strong correlation between the two methods [27]. Cobb and colleagues also emphasized that a digital photograph-based measurement technique quantified foot posture parameters consistently and reproducibly, offered significant advantages over traditional clinical methods, and provided high reliability even with inexperienced raters [28]. In a more recent study, it was shown that foot arch measurements obtained using a simple photographic technique did not differ meaningfully from those obtained by conventional footprint analysis and could even detect early, small-magnitude pes planus and pes cavus deformities [29]. Another study reported that, in adult flatfoot, standardized rearfoot photographs and Harris–Beath mat imprints showed significant relationships with multiple radiographic angles [19]. The potential of smartphone cameras in screening for pes planus has also been demonstrated in various investigations: for example, in a study by Ghandour and colleagues integrating a smartphone camera with a deep learning algorithm, the diagnosis of pes planus was made with high accuracy (sensitivity 87% and specificity 84%; AUC 0.90), indicating that this method may be a reliable and easily accessible screening tool [18]. Similarly, Eksen and colleagues reported that a mobile application they developed produced results that were 92% concordant with orthopedic specialist evaluations by analyzing patient foot images, demonstrating that smartphone-based analyses may be successful in the preliminary diagnosis of flatfoot [30]. The correlation between clinical/photographic alignment metrics and radiographic alignment in adult flatfoot deformity has also been prospectively confirmed; moreover, it has been shown that camera angle (0°, 20°, 40°) has no significant effect on clinical readings [31]. Our findings likewise show that high diagnostic accuracy for pes planus classification (AUC 0.885 in our study) can be achieved with calibrated photographic measurements, supporting the use of smartphone-based foot arch assessment as a radiation-free method, particularly for early screening and follow-up. Considering all this data, the existing literature and our own results confirm that smartphone- and photograph-based measurement techniques stand out as reliable, practical, and effective screening tools for the evaluation of pes planus. Nevertheless, relatively few studies have directly compared smartphone-based photographic metrics with radiographic standards in the same individuals for the same angles; where comparative work exists, it more commonly links clinical/footprint or photographic measures to radiographs in broader terms rather than performing one-to-one smartphone–radiograph angle validation [25].

In addition to the P-APA measurement that is the focus of our study, footprint-based screening methods have also been widely used and supported in the evaluation of pes planus. In adult flatfoot, Harris–Beath mat prints have shown significant relationships with clinical heel valgus and multiple radiographic angles, and have been validated as an effective method for quantifying deformity in a simple, inexpensive, and reproducible manner [19]. In childhood, the Staheli Arch Index (AI) obtained from the Harris–Beath mat has been used in combination with lateral radiographic measurements to define the medial longitudinal arch and has been proposed as a low-cost, non-invasive approach for screening and outpatient use. Likewise, footprint indices such as the Chippaux–Smirak index (ratio of metatarsal width to arch narrowing) and Clarke’s angle (angle measurement based on medial border concavity) are low-cost, non-invasive screening options and carry meaningful diagnostic value in pronounced flatfoot [12,32,33]. The Visual Arch Index (Visual-AI), which is a practical and rapid visual categorization tool derived from the area-based numeric AI, offers rapid classification in the clinic with high rater agreement; protocols for the Foot Posture Index-6 (FPI-6) (including those using five photograph-based criteria) have been reported to yield excellent intra-rater and good inter-rater reliability and to correlate significantly with radiographic angles in pronounced adult flatfoot [20,34]. A newer approach, the “simple footprint assessment board” system, has also reported significant correlations with the computed tomography (CT)-based navicular index and with tibiocalcaneal and calcaneal pitch angles, and reliability around ICC 0.93, demonstrating that flatfoot can be screened without radiation and in a manner generalizable across age groups [30,33]. When these data are considered together, although radiography remains the most widely used imaging reference in clinical practice, photographic and footprint-based methods can be safely used for screening, epidemiologic research, and follow-up, provided standardized protocols and calibration are used [24,35]. The recently added guideline text that “defines radiologic measurements” strengthens the standardization of reporting of our findings by clarifying the terminology and measurement definitions of clinical and radiographic parameters [24], and a methodological study on the FPI and related measurements offers practical principles regarding measurement theory and standardization of implementation [36]. From a methodological standpoint, future studies should adopt a multiparametric panel that integrates CPA with sagittal radiographic measures (e.g., Meary’s and talo-horizontal angles) and validated clinical indices (FPI-6, AI) and externally validate calibration equations across age strata and deformity severity. Emerging low-cost imaging platforms—such as red–green–blue–depth (RGB-D) camera systems and smart footprint sensors—already report very low errors and high repeatability, offering a feasible route to standardized, radiation-free screening pipelines that interface seamlessly with smartphone workflows [37,38].

Certain limitations of this study should be considered. First, our sample size was relatively small, and validation in a larger, multicenter patient population could improve generalizability. In addition, since our sample was limited to skeletally mature adults, the findings may not be directly generalizable to pediatric or elderly populations. Moreover, because all participants were symptomatic patients evaluated in a tertiary orthopedic clinic, spectrum bias may limit the generalizability of our findings to asymptomatic individuals or primary care and community settings. Because the angle used in photographic measurements is based solely on calcaneal inclination, the multidimensional nature of pes planus (for example, medial arch collapse, midtarsal joint mobility, forefoot abduction) could not be fully reflected. Small deviations due to human factors may occur in measurements; therefore, inter-observer standardization and the integration of digital analysis software are important for future studies. Also, the calibration formula used may not achieve the same level of precision for every individual. However, Deming regression reduced the average error; a certain margin of error persists in individual measurements. This may limit the use of photographic data alone for diagnostic purposes, especially in borderline cases. Another limitation is that the calibration equation and ROC-based cutoff were derived and evaluated in the same cohort, which may introduce optimism bias; therefore, independent validation (e.g., cross-validation/bootstrapping) or an external cohort is required before the model and cutoff can be considered for routine screening or telemedicine implementation. Finally, our comparison with radiography, which is a widely used imaging reference for the structural assessment of pes planus, was limited to lateral radiographs, and other radiographic angles (for example, hindfoot alignment and talonavicular coverage) were not evaluated. For these reasons, the results of our study should be interpreted with caution. In future research, the robustness of the findings should be tested by including multidimensional imaging methods and larger populations [39]. In line with these limitations, combining calibrated P-APA with dynamic footprint or pressure-based indices may capture function-related arch behavior while avoiding repeated radiation exposure. It could strengthen longitudinal monitoring in primary care and telemedicine [25].

## 5. Conclusions

This study showed that the photographic arch pitch angle (P-APA) measured from standardized smartphone photographs is highly reliable; however, it is a non-equivalent surrogate of radiographic calcaneal pitch and systematically overestimates radiographic values, with limited agreement at the individual level. Therefore, smartphone photogrammetry cannot replace weight-bearing radiographs, and conventional radiography remains the reference standard for diagnosis and clinical decision-making. Smartphone-based assessment may be used only as a complementary tool for preliminary screening or telemedicine support [40]; any positive or equivocal findings should be confirmed with weight-bearing radiographs. Although calibration may improve diagnostic discrimination in a derivation cohort, the calibration model and cutoff must be independently validated before clinical deployment.

## Figures and Tables

**Table 1:** Inter-observer Reliability (ICC (2,1) with 95% CI).

Modality	Session	ICC (2,1)	95% CI	Interpretation
Photograph	*Time 1*	0.971	0.960–0.979	Excellent
	*Time 2*	0.980	0.972–0.986	Excellent
Radiograph	*Time 1*	0.989	0.985–0.992	Excellent
	*Time 2*	0.993	0.990–0.995	Excellent

Abbreviations: ICC: Interclass Correlation Coefficient, CI: Confidence Interval.

**Table 2:** Intra-observer Reliability (ICC (3,1) with 95% CI).

Rater	Modality	Measures	ICC (3,1)	95% CI	Interpretation
A	Photograph	*Time 1 vs. 2*	0.971	0.959–0.980	Excellent
	Radiograph	*Time 1 vs. 2*	0.988	0.983–0.992	Excellent
B	Photograph	*Time 1 vs. 2*	0.973	0.960–0.982	Excellent
	Radiograph	*Time 1 vs. 2*	0.99	0.985–0.993	Excellent
C	Photograph	*Time 1 vs. 2*	0.977	0.967–0.984	Excellent
	Radiograph	*Time 1 vs. 2*	0.989	0.985–0.992	Excellent

Abbreviations: ICC: Interclass Correlation Coefficient, CI: Confidence Interval.

**Table 3:** Diagnostic classification of pes planus using the calibrated photographic cutoff (≤22.8°) with radiographic CPA < 18° as reference standard.

	Radiographic Pes Planus (CPA < 18°)	Radiographic Normal (CPA ≥ 18°)	Total
Test positive (calibrated ≤ 22.8°)	28	28	56
Test negative (calibrated > 22.8°)	0	44	44
Total	28	72	100

Sensitivity 100% (95% CI, 87.7–100.0); specificity 61.1% (95% CI, 48.9–72.4); PPV 50.0% (95% CI, 36.3–63.7); NPV 100.0% (95% CI, 92.0–100.0). CIs were calculated using the exact binomial method.

## Data Availability

The datasets are not publicly available. The de-identified data are available upon request to the corresponding author due to privacy, ethical, and legal restrictions protecting patient confidentiality.

## Abbreviations

The following abbreviations are used in this manuscript:

CPA	Calcaneal Pitch Angle
P-APA	Photographic Arch Pitch Angle
BMI	Body Mass Index
ICC	Intraclass Correlation Coefficient
CI	Confidence Interval
SD	Standard Deviation
LoA	Limits of Agreement
ROC	Receiver Operating Characteristic
AUC	Area Under the Curve
CCC	Concordance Correlation Coefficient
RMSE	Root Mean Squared Error
MAE	Mean Absolute Error
PI	Prediction Interval
PACS	Picture Archiving and Communication System
FPI-6	Foot Posture Index-6
AI	Arch Index
Visual-AI	Visual Arch Index
